# Minor surgery in primary care has reduced minor surgery waiting lists: a 12-month review

**DOI:** 10.1007/s11845-022-02928-9

**Published:** 2022-02-04

**Authors:** Darren McCormack, Alexandra Frankel, Joe Gallagher

**Affiliations:** Department of General Practice, Trinity College Dublin, Dublin, Ireland

**Keywords:** Community surgery, Minor surgery, Plastic surgery, Skin cancer

## Abstract

**Background:**

To investigate minor procedures performed in general practice, to alleviate the burden on the economy, patients and secondary care physicians.

**Aims:**

To determine the range of minor surgical procedures performed in a single group general practice, subsequent referrals made to secondary care, follow-up surgical procedures required and post-operative complications within a patient group.

**Methods:**

Retrospective data collection from the electronic records of a single group general practice consisting of 5101 active patients within the previous 12 months. Through use of Socrates software program and statistical analysis, patients were stratified into demographics, including, age, gender, the cost of the procedure, the type of procedure being carried out, the post-operative referral rate to secondary care and any subsequent procedures required. The patients were excluded if their minor procedure was classified as a joint injection.

**Results:**

133 procedures were carried out over the 12-month period. Of these patients, the majority were male, and the mean age was 44 years old. The most common procedures included the removal of ingrown toenails, lesion excisions and punch biopsies. Histological analysis was done on biopsies, with a low rate of misdiagnosis pre-operatively. Additionally, there were minimal referrals and no complications recorded.

**Conclusions:**

This study has demonstrated the ability for minor surgery to be safely carried out in primary care. The integration of general practice, general surgery and plastic surgery could provide a higher level of patient care and exchange of skills to help reduce waiting lists and alleviate the burden secondary care.

## Introduction

In Ireland, it is thought that community surgical services will form a vital role in providing simple and minor surgical procedures in the future. There is a focus on transferring appropriate hospital-based workload to community healthcare providers under the Sláintecare Act [1]. This proposal would aid with the fallout of cancellations of elective and minor surgical appointments following the COVID-19 pandemic and the resultant increased waiting list times [2]. Additional support from community surgical services has been proposed, to help alleviate the pressure on secondary care and allow surgical specialities to focus on procedures that cannot be provided elsewhere and require immediate attention [3]. There has been a trend in general practice towards evolving services provided and utilising the skills in primary care. This would in turn augment and support the care provided by secondary care [4]. Currently, many minor procedures, such as punch biopsies and basic non-melanoma skin surgeries, are carried out in a fully staffed surgical theatre, increasing the burden on secondary care physicians. These minor surgeries could easily be delivered within primary care and within an outpatient setting. Australia has a well-established community surgery service providing these procedures, as well as strong integration between general practice, surgery, and the skin multi-disciplinary team [5]. This integration ensures these procedures are carried out safely and successfully within the community, while providing a way of relieving the pressure on surgical  waiting lists.

This study aims to investigate how these procedures can be carried out in a well-funded general practice in Ireland, decreasing both the burden financially on the healthcare system, as well as logistically by minimizing wait lists and demand on surgery clinics. This would allow the plastic/general surgical speciality to focus on increasingly urgent and complicated procedures requiring a secondary care setting. This study looks to explore the range of minor surgical procedures performed in a primary care general practice setting, subsequent referral to secondary care, follow up surgical procedures performed in secondary care and post-operative complications associated with the procedure. Overall, the aims of this study were to determine the gender, age and body location of the minor procedures being carried out in general practice, the type of procedure being carried out. Additionally, if the pre-operative diagnosis matches the diagnosis after histology results were received, and finally, the referral rate to secondary care and subsequent procedures and complications.

## Materials and methods

This is a retrospective cohort study examining minor surgery in general practice and its effect on surgery wait lists over 12 months. Data was derived from the electronic records of a single group general practice in Co. Wexford, Ireland. The electronic records of 6 general practitioners were reviewed and examined, consisting of 5101 active patients. Clinical records were accessed with Socrates software program and statistical analysis carried out. Statistical analysis was used to determine the demographics in which the procedure was being carried out, the cost of the procedure, the type of procedure and the referral rate to secondary care, including any subsequent procedures required.

A patient list was created, consisting of all patients who attended the general practice for minor procedures within the previous 12 months. It was then determined which patients were eligible within the patient group for statistical analysis. Exclusion criteria consisted of minor procedures, solely consisting of joint injections. Inclusion criteria were any patient within the general practice who underwent a minor procedure, other than a joint injection, within the 12 months prior. Clinical records were examined for this patient group and data was extracted, looking at “Gender”, “Age”, “Date Performed”, “Type of Procedure”, “Location on Body”, “If Sent for Histology”, “Payment Type”, “Pre-/Post-op Diagnosis”, “Referral to Secondary Care”, and “Further Surgical Intervention”.

## Results

One hundred thirty-three minor procedures were carried out over the 12-month period within the general practice. The mean age was 44 years old with an age range from 9 to 84 years of age. The distribution of gender for the procedures was 44% female and 56% male. Thirty-six percent of the procedures were the removal of an ingrown toenail. Seventeen percent of the cases were excision of a lesion, and the remaining 12% of cases consisted of punch biopsies. The most common procedures performed in the primary case setting consisted of removal of an ingrown toenail (46 cases), lesion excisions (32 cases) and punch biopsies (17 cases). Of the biopsies taken, 58 cases were sent for histological analysis. In comparing pre- and post-operative diagnoses, 12 cases underwent a change in diagnosis following histopathology. In 5 cases the pre-histology diagnosis was changed from malignant to benign, and in 1 case it was changed from benign to malignant. Eight cases over the 1-year period required further hospital referral for surgery, and no post-operative complications were recorded from general practice, demonstrating safe and appropriate surgical patient care.

## Discussion

From this study, it can be determined that minor surgery can be safely carried out in a primary care setting, with minimal need for secondary care referral and follow-up procedures. Of the total biopsies taken in the community setting, there was a low rate of misdiagnosis as evidenced by histopathology results in this study. The integration of primary care and surgical departments could provide an increased quality of patient care and exchange of skills. This would contribute to the reduction of waiting lists and removal of inappropriate procedures from the secondary care setting.

## Conclusion

The National Cancer Control Programme in collaboration with the Irish College of General Practitioners has produced a draft template for the integration of general practice-based care into a coordinated service for non-melanoma skin cancer. With an excess of 10,000 incident cases annually as well as even more pre-malignant lesions and concerned patients, an integrated service between plastic surgery, general surgery and primary care may provide a solution to the waiting list burden on patients, increasing healthcare costs and increasing workload burden on secondary care physicians. The future vision for the health service is aimed at delivering community-based services, where appropriate, to relieve the pressure on secondary care. We have shown how minor surgical services can be safely and effectively delivered in a community setting, providing a simple and cost effective solution for these patients.
